# Anticipatory competence of adolescents with movement disorders in the prevention of deviations

**DOI:** 10.1192/j.eurpsy.2024.1369

**Published:** 2024-08-27

**Authors:** A. Akhmetzyanova, T. Artemyeva

**Affiliations:** Department of Psychology and Pedagogy of Special Education, Kazan Federal University, Kazan, Russian Federation

## Abstract

**Introduction:**

Anticipation is the most valuable component of the regulatory side of human behavior. Adolescence is a sensitive period in relation to the formation of an anticipatory and prognostic system, which in turn provides an opportunity to assess causal relationships and evaluate the consequences of actions taken.

**Objectives:**

Study of the anticipatory viability of adolescents with long-term disorders in the prevention of deviations.

**Methods:**

The study involved 46 adolescents aged 11-15 studying at a specialized boarding school for children with disabilities. The observational method was used as well as the author’s methodology “Studying the anticipatory solvency of adolescents” Akhmetzyanova A.I., Artemyeva T.V.; “Diagnostic questionnaire for identifying propensity to various forms of deviant behavior for students of educational institutions” developed by the Department of Psychiatry of the Military Medical Academy named after S.M. Kirov.

**Results:**

The subjects had difficulty predicting the passage of time, with its adequate and rational distribution, including planning their own activities. Adolescents with musculoskeletal disorders had difficulty making a pragmatic and realistic forecast of possible events in communication with other people, as well as predicting the emotional states of interaction participants. Adolescents with movement disorders were characterized by an inadequate assessment of themselves as a subject of professional activity, fixation on the movement disorder, and high levels of anxiety and neuroticism. The subjects showed a tendency to suicidal behavior due to risk factors such as high levels of anxiety associated with self-esteem and anxiety in interpersonal relationships, high affectivity and demonstrativeness, social pessimism and negative prediction of the future. During the correlation analysis, the relationship between spatio-temporal and speech-communicative anticipatory consistency with indicators of deviant behavior - delinquent behavior and deviant behavior was revealed.

**Conclusions:**

The data obtained in the study will allow specialists to timely identify and prevent the development of deviant behavior, as well as build a route for correctional classes with each child.. This paper has been supported by the Kazan Federal University Strategic Academic Leadership Program.

**Disclosure of Interest:**

None Declared

